# Identification of Three Novel O-Linked Glycans in the Envelope Protein of Tick-Borne Encephalitis Virus

**DOI:** 10.3390/v16121891

**Published:** 2024-12-08

**Authors:** Ebba Könighofer, Ekaterina Mirgorodskaya, Kristina Nyström, Karin Stiasny, Ambjörn Kärmander, Tomas Bergström, Rickard Nordén

**Affiliations:** 1 Department of Infectious Diseases, Institute of Biomedicine, Sahlgrenska Academy, University of Gothenburg, 413 46 Gothenburg, Sweden; 2 Proteomics Core Facility, Sahlgrenska Academy, University of Gothenburg, 405 30 Gothenburg, Sweden; 3 Center for Virology, Medical University of Vienna, 1090 Vienna, Austria; 4 Department of Clinical Microbiology, Sahlgrenska University Hospital, 413 46 Gothenburg, Sweden

**Keywords:** E protein, N-linked glycan, O-linked glycan, tick-borne encephalitis virus

## Abstract

The tick-borne encephalitis virus is a pathogen endemic to northern Europe and Asia, transmitted through bites from infected ticks. It is a member of the *Flaviviridae* family and possesses a positive-sense, single-stranded RNA genome encoding a polypeptide that is processed into seven non-structural and three structural proteins, including the envelope (E) protein. The glycosylation of the E protein, involving a single N-linked glycan at position N154, plays a critical role in viral infectivity and pathogenesis. Here, we dissected the entire glycosylation profile of the E protein using liquid chromatography-tandem mass spectrometry and identified three novel O-linked glycans, which were found at relatively low frequency. One of the O-linked glycans was positioned close to the highly conserved N-linked glycan site, and structural analysis suggested that it may be relevant for the function of the E 150-loop. The N154 site was found to be glycosylated with a high frequency, containing oligomannose or complex-type structures, some of which were fucosylated. An unusually high portion of oligomannose N-linked glycan structures exhibited compositions that are normally observed on proteins when they are translocated from the endoplasmic reticulum to the trans-Golgi network, suggesting disruption of the glycan processing pathway in the infected cells from which the E protein was obtained.

## 1. Introduction

The tick-borne encephalitis virus (TBEV) is a pathogen found in large parts of northern Europe and Asia. It is transmitted via bites from infected ticks and can cause systemic and neurological infections with mortality rates ranging from >2% to 34% depending on subtype [1,2]. Available vaccines protect against severe disease; however, booster doses are recommended every three to five years for healthy individuals below 50 years of age, while elderly and immunosuppressed individuals require a more extended vaccine program. Rare breakthrough infections and vaccine failures occur despite adherence to the vaccination schedule [3,4,5].

TBEV belongs to the family *Flaviviridae*, which consists of small, enveloped viruses with positive-sense, single-stranded RNA genomes. The TBEV genome translates to a polypeptide that is further processed into seven non-structural and three structural proteins: the capsid (C) protein, the membrane (M) protein, and the envelope (E) protein [6,7]. The E protein consists of three domains: domain I (ED I), which stabilizes the protein and contain the E 150-loop [8,9]; domain II (ED II), which contains a conserved fusion loop essential for membrane fusion [10]; and domain III (ED III), which has an immunoglobulin-like fold and serves as a structural target for neutralizing antibodies. Three linear B-cell epitopes have been identified on the E protein: one located in ED I (aa 162–179) and two in ED II (aa 51–58 and 222–239) [11]. Structural antibody epitopes, comprising discontinuous segments of the E protein or comprising residues from nearby E proteins in the quaternary structure, are also present [12,13,14].

Starting at N154, the ED I of TBEV E protein contains one conserved consensus site (N-X-T/S (X≠P)) for N-linked glycosylation. The N-linked glycan at N154 has been connected to important functions in viral entry, infectivity, and pathogenesis of TBEV as well as in other flaviviruses [15,16,17,18,19]. Despite its importance, few studies have determined the structure of the N-linked glycan [20]. The E protein also contains 67 sites (serine and threonine) for potential O-linked glycosylation, but to our knowledge, there are no data showing O-linked glycosylation. Consequently, we mapped the glycans of the E protein from TBEV grown in human adenocarcinomic alveolar basal epithelial (A549) cells. We identified three novel O-linked glycans and determined the composition of the N-linked glycan structures at position N154 on the E protein.

## 2. Materials and Methods

### 2.1. Cells and Virus

The human adenocarcinomic cell line A549 (A549-CCL-185) was obtained from ATCC and cultured in 150 cm^2^ cell culture flasks containing 28 mL Eagle’s Minimal Essential Medium (Thermo Fisher Scientific, Waltham, MA, USA) supplemented with 8% fetal calf serum and 1% penicillin-streptomycin until 100% confluence. Then, 1 mL aliquot of virus stock prepared in A549-cells (TBEV originally isolated from the blood of a Swedish TBE patient and classified as a European subtype [21], 50% tissue culture infectious dose (TCID50) 10^6^/mL) was added to the cells, and the infection was allowed to proceed for 48–72 h. Extracellular viral particles were harvested by removing the cell culture media from the infected cells, after which the virus-containing cell culture media was concentrated using density gradient centrifugation. First, cellular debris was removed by centrifugation at 5000× *g* for 10 min at 4 °C. The supernatant was transferred to QuickSeal Ultracentrifuge Tubes (Beckman Coulter, Indianapolis, IN, USA) and centrifuged at 100,000× *g* for 1 h at 4 °C. Proteins in the supernatant were concentrated using the Macrosep Centrifugal Device with a 10 K MWCO (Pall, Port Washington, DC, USA). The samples were loaded twice onto the centrifugal device and centrifuged at 4200× *g* for 30–60 min at 4 °C. The membrane was washed with Tris-buffered saline (TBS), and the retentate was collected and transferred to Eppendorf tubes for storage at −20 °C prior to E protein purification using immunoaffinity columns. The amplification of TBEV in cell culture was performed at our Biosafety Level 3 (BSL-3) facilities at the Department of Clinical microbiology at the Sahlgrenska University hospital.

Intracellular viral particles and cells were lysed in 8 mL lysis buffer (Tris phosphate-buffered saline (TBS) supplemented with 2% 3α,12α-dihydroxycholanic acid sodium salt (DOC), 2% Nonidet P-40, and 1 mM Pefabloc^®^ SC (4-(2-aminoethyl)benzenesulfonyl fluoride hydrochloride, AEBSF)), followed by douncing in a 12 mL glass douncer on ice for 1 h before purification of the E protein using immunoaffinity columns.

### 2.2. Purification of E Protein Using Immunoaffinity Column

Coupling of monoclonal antibodies (mAb B2 targeting TBEV E protein DIII) [12] to CNBr-sepharose was performed by the swelling of 1 g sepharose (GE Healthcare, Chicago, IL, USA) in 1 mM HCl for 15 min during vacuum suction in a Büchner fennel, followed by rinsing with coupling buffer (0.1 M NaHCO_3_ and 0.5 M NaCl, pH 8). Then, 0.5 mL sepharose was transferred to a silicon tube, and 0.9 mg monoclonal anti protein E antibody was added to the tube and allowed to bind during end-to-end rotation for 2 h at room temperature. The tube was centrifuged 5 min, 2000× *g* at room temperature and the supernatant removed. Blocking buffer (0.2 M glycine, pH 8) was added in a volume equal to the gel and the tube again incubated 2 h during end-to-end rotation. The centrifugation was repeated and the supernatant removed, followed by interchangeable washing and centrifugation with acetate buffer (0.1 M CH_3_COONa and 0.5 M NaCl, pH 4) and coupling buffer 5 times. The final wash was performed with coupling buffer. The sepharose-coupled antibodies were stored at 4 °C in 20% EtOH in phosphate-buffered saline (PBS) until use. The sepharose-coupled antibody column was washed with 20 mL wash buffer with detergent (0.01 M Tris-HCl, 0.5 mM NaCl, and 0.1% Nonidet P-40). The solution containing solubilized extracellular and intracellular viral particles was applied to the column and allowed to flow through. To maximize E protein binding, the flow through was allowed to pass through the column once more before washing the column with 20 mL wash buffer with detergent, followed with washing with 20 mL wash buffer without detergent (0.01 M Tris-HCl and 0.5 mM NaCl). The E protein captured by the column was eluted by addition of elution buffer (0.1 M glycine-HCl, pH 2.6) in 1 mL fractions. Next, 100 µL neutralization buffer (1 M Tris, pH 8) was added to each fraction to reestablish neutral pH in the sample. The binding to and washing and elution of the E-protein from the immunoaffinity column was performed at 4 °C.

The protein concentration in each fraction was measured using the Pierce BSA Protein Assay Kit (Thermo Fischer Scientific, Waltham, MA, USA) according to the instructions from the manufacturer and the absorbance measured at 492 nm.

### 2.3. NanoLC MS Analysis

Purified E protein fraction of 1 mL at an estimated concentration of 1 µg/mL was processed using the modified filter-aided sample preparation (FASP) method [22]. In brief, the E protein fraction was reduced with 100 mM Dithiothreitol (DTT) at 60 °C for 30 min, transferred to 30 kDa MWCO Pall Nanosep centrifugation filters (Sigma-Aldrich), and washed several times with 8 M urea and once with digestion buffer (50 mM triethylammonium bicarbonate buffer (TEAB) and 0.5% sodium deoxycholate (SDC)) prior to alkylation with 18 mM 2-iodoacetamide (IAM) for 30 min at room temperature (in the dark). Samples were digested with 0.1 µg trypsin (Pierce MS grade Trypsin, Thermo Fisher Scientific, ratio 1:10) at 37 °C overnight, followed by an extra 0.1 µg trypsin addition and 2 h incubation at 37 °C. The produced proteolytic peptides were collected by centrifugation, and filters were additionally washed with 50 mM TEAB to ensure that all material eluted from the filters. The collected proteolytic peptides were first purified using HiPPR™ Spin Column, followed by acidification with 10% TFA and subsequent centrifugation to remove remaining SDC. The SDC-free supernatant was desalted using Pierce Peptide Desalting Spin Columns (Thermo Scientific) prior to LC-MS/MS analysis.

The E protein proteolytic preparation was analyzed on a QExactive HF mass spectrometer interfaced with the Easy-nLC1200 liquid chromatography system (ThermoFisher). Peptides were trapped on an Acclaim Pepmap 100 C18 trap column (100 µm × 2 cm, particle size 5 µm; Thermo Fisher) and separated on an in-house packed analytical column (75 µm × 30 cm, particle size 3 µm, Reprosil-Pur C18; Dr. Maisch, Ammerbuch-Entringen, Germany) using a gradient from 5% to 35% acetonitrile in 0.2% formic acid over 75 min at a flow of 300 nL/min. Each preparation was analyzed using two different MS1 scans settings, i.e., in the *m*/*z* range of 375–1500 and 600–2000, both at a resolution of 120 K. MS2 analysis was performed in a data-dependent mode at a resolution of 30 K, using a cycle time of 3 s. The most abundant precursors with charges 2–7 were selected for fragmentation using HCD at a normalized collision energy setting of 28. The isolation window was set to either *m*/*z* = 1.2 for data acquired in the *m*/*z* range of 380–1500 or *m*/*z* = 3.0 for data acquired in the *m*/*z* range of 600–2000. The dynamic exclusion was set to 10 ppm for 20 s.

### 2.4. Database Search and Data Processing

The acquired data were analyzed using Proteome Discoverer version 2.4 (Thermo Fisher Scientific). Database searches were performed with either Byonic (Protein Metrics) or Sequest as search engines. The data were searched against custom database consisting of SwissProt_human database (20,342 proteins) and the sequence of purified E protein. First, the data were searched with Sequest to evaluate the E protein preparation purity. After that, all glycoproteomic searches were carried out with Byonics (Protein Metrics) using the selected N- and O-glycan database (186 and 30 glycan compositions, respectively). The precursor mass tolerance was set to 5 ppm (Sequest) or 10 ppm (Byonics) and fragment mass tolerance to 30 ppm. Tryptic peptides with up to two missed cleavages were accepted. Variable modification of methionine oxidation and fixed cysteine alkylation were allowed. Target Decoy was used for Peptide Spectrum Match (PSM) validation. Prior to the final assignment, the identified glycosylated peptides were manually evaluated based on the observed fragmentation pattern, the number of glycoforms per site, number of PSM per glycoform, and the retention time windows for the different observed glycoformes (at the same site). The extracted ion chromatogram (EIC) peak intensities were used to determine the glycoform abundances, expressed as the percent of the total signal for all modified and non-modified peptides sharing the same amino acid sequence. Only structures with a relative abundance of ≥1% are listed and shown in figures and tables.

### 2.5. In Silico Glycosylation

The crystal structures of the E-protein (PDB ID 7QRE and 1SVB) were downloaded from Research Collaboratory for Structural Bioinformatics Protein Data Bank (RCSB PDB). Heteroatoms and the precursor membrane protein fragment of 7QRE were removed using ChimeraX [23]. The attachment of glycans as well as the calculation of the solvent accessible surface area were performed by uploading the structures to the glycoprotein builder from GLYCAM Web [24]. The condensed glycan notations of the attached glycans are as follows:

O-linked glycans (residues T76, T81, T147, S158, and S285): DGalpNAca1-OH N-linked glycans (residues N154): DManpa1-6[DManpa1-3]DManpa1-6[DManpa1-2DManpa 1-3]DManpb1-4DGlcpNAcb1-4DGlcpNAcb1-OH

## 3. Results and Discussion

**Figure 1:**
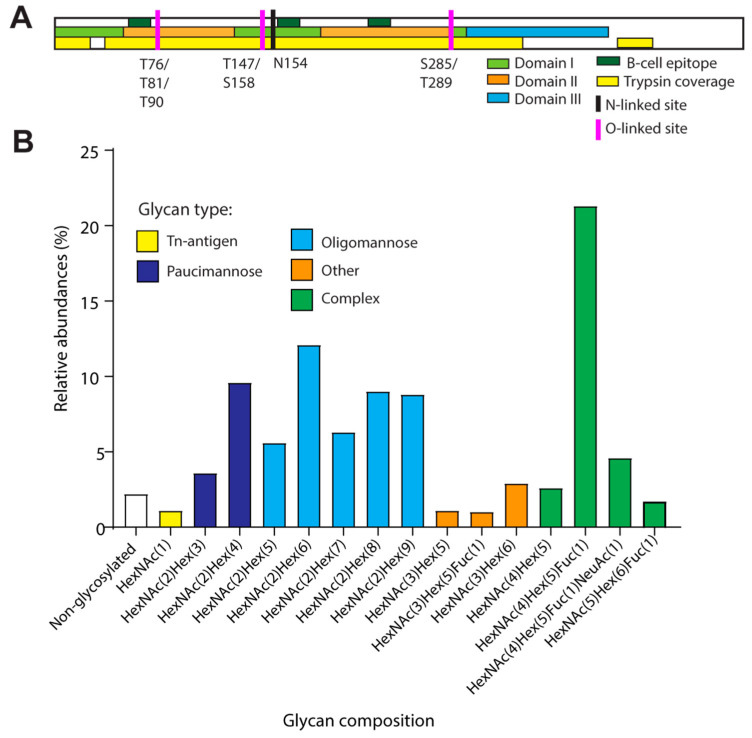
The positions and structures of the O- and N-linked glycans within the E protein of TBEV. (**A**) Schematic drawing of the E protein, showing the B cell-epitopes identified by Kuivanen S et al. [11], domains D I–III, the trypsin coverage used in our glycoproteomic analysis, and the identified sites for N-and O-linked glycosylation. (**B**) Glycoform distribution of identified structures detected on the peptide covering the N-linked glycosite. Structures classified in the category “other” include both N-linked glycans of hybrid type and a combination of N-linked and O-linked glycoforms present on the same peptide. Structures with a relative abundance of ≤1% are excluded.

TBEV was grown in A549 cells, and extra- and intracellular viral particles were harvested and lysed before purification of the E protein on an immunosorbent column coated with a monoclonal antibody targeting an epitope within ED III. Proteolytic cleavage of the isolated E proteins with trypsin resulted in a peptide coverage of 70%. The resulting peptides were analyzed for glycan modifications using liquid chromatography tandem-mass spectrometry (LC-MS/MS) (Figure 1A). The peptide coverage included the linear B-cell epitopes [11], the consensus site for N-linked glycosylation N154-T156, and 54 out of 67 possible sites (80%), i.e., serine (S) or threonine (T), for O-linked glycosylation.

The N-linked consensus site N154-T156 was highly occupied, with 96.7% of the peptides carrying N-linked glycan modifications at N154. Multiple glycoforms were observed, and those above 1% of the total for the peptide V143-R160 are shown in Figure 1B and Table S1. Of these glycoforms, 42% carried an oligomannose-type glycan (Figure 1B), with Man_6_GlcNac_2_ being the most abundant, present on 12% of analyzed peptides. High degrees of oligomannose structures have been observed in proteins such as the human immunodeficiency virus (HIV) envelope protein gp120, which might indicate a low degree of glycan processing [25]. In gp120, the densely clustered N-linked glycans sterically hinder enzymatic elongation; however, the oligomannose structures of the single N-linked glycan of the E proteins might instead be important for correct protein folding during maturation [26] or for interactions with receptor proteins, thereby regulating infectivity and pathogenesis [15,16,17,18,19]. Interestingly, a large fraction of the oligomannose structures contained -8 or -9 mannose residues. These types of structures are the major glycoforms prior to exit from the endoplasmic reticulum (ER) during glycan synthesis and are subsequently trimmed down in the trans-Golgi network (TGN) before elongation and generation of complex type N-linked glycan structures [27].

During the sample preparation, we harvested both intra- and extracellular viral particles prior to the isolation of the E protein. Thus, it is possible that a significant part of the isolated E protein was captured before it was fully processed by the glycosylation machinery. We did not calculate the ratio between intra- and extracellular viral particles before isolating the E protein, and it was not possible to determine if there is a selection bias regarding the glycosylation status of the E protein within the immunosorbent column due to the limited amount of isolated E protein. The intracellular fraction is likely to consist of a spectrum of E protein, ranging from immature, not glycosylated protein to fully processed protein. To define the glycan profile with higher resolution, it would be necessary to fractionate the TBEV-infected cells to separate the cellular compartments before isolating the E protein prior to LC-MS/MS. In addition, flavivirus particles show considerable heterogeneity upon release from the infected cell, both in terms of the maturation state and internal protein architecture [28]. This suggests that the protein maturation process within the ER and the TGN may be subject to considerable disruptions that could impact glycosylation.

Complex-type N-linked glycan structures were found on 30% of the peptides, mainly of the composition HexNac_4_Hex_5_Fuc_1_ (present on 21% of the analyzed peptides) (Figure 1B). A fucose group was identified on 28% of the complex-type glycans, and manual inspection of the MS spectra suggests core fucosylation of the innermost GlcNAc. The addition of a core fucose is catalyzed by the enzyme fucosyltransferase 8 (FUT 8), which has been shown to be upregulated in hepatitis B virus (HBV)-transfected hepatoma cells [29,30]. Overexpression of FUT8 has been correlated with increased binding of HBV-like particles to cells [30]. Since viral infection can alter host cell gene expression to promote glycan compositions favorable for viral spread and replication, the high degree of core fucosylation might enhance the infectivity of TBEV [31,32,33].

Only 5% of the N-linked glycans carried one or more sialic acid residues, indicating limited access by sialyltransferases. Sialic acid plays an important role in pathogen recognition by macrophages via the sialic acid-binding immunoglobulin-like lectin sialoadhesin (Siglec-1), found on certain macrophage subsets [27]. Siglec-1 primarily recognizes α2-3 linked sialic acids, and binding leads to increased uptake of viral particles and enhanced infection, as shown for HIV [27,34]. Since TBEV infects macrophages, a possible connection to Siglec-1 warrants further investigation [35].

Thirteen percent of the peptides covering the N-linked consensus site carried N-linked glycans of paucimannose type (Man_3-4_GlcNAc_2_(Fuc_1-2_)). Paucimannose typically decorates the E protein when the virus is grown in tick cells [20] and is commonly observed in invertebrates. Vertebrates, however, typically extend the glycan precursor into complex-type N-linked glycans [36]. Previous studies have also identified paucimannosidic structures in tumor cells [37,38], which could explain our findings. Almost 5% of the N-linked glycans were assigned as “other” in Figure 1B. These structures may comprise either hybrid-type structures or a combination of N- and O-linked structures present on the same peptide. PNGaseF-treatment prior to reanalysis would remove the N-linked glycans, thereby enabling identification and analysis of O-linked glycoforms. However, the limited quantity of purified E protein at our disposal prevented further differentiation of these glycoforms.

**Figure 2:**
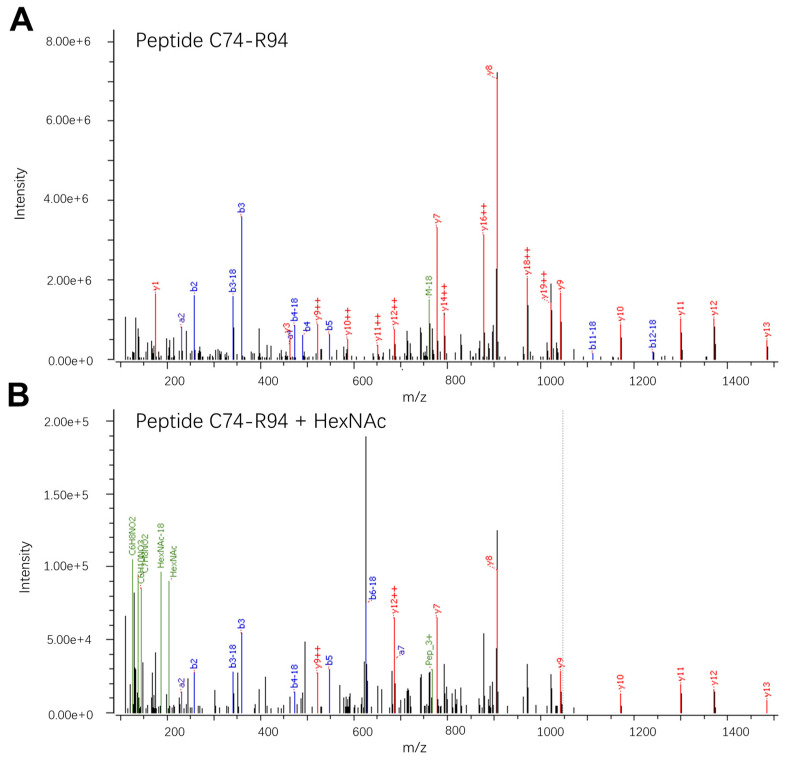
LC-MS/MS spectra showing the fragmentation patterns of the peptide C74-R94: CPTMGPATLAEEHQGGTVCKR, MW = 2299.0508 Da. (**A**) Fragment ion spectrum of the non-glycosylated peptide; *m*/*z* = 767.3571; z = 3. (**B**) Fragment spectrum of the corresponding peptide carrying a single HexNAc modification; *m*/*z* = 626.5398; z = 4. The observed peptide fragments (b and y) are marked as well as the glycan oxonium ions observed in the low-*m*/*z* region of the fragment spectrum of the HexNAc-modified peptide.

Three O-linked glycans were identified on the E protein (Figure 1A). First, manual inspection of the fragment ion spectra suggests a single HexNAc monosaccharide present in the region covering amino acids C74-R94, containing three potential sites for O-linked glycosylation (T76, T81, and T90) (Figure 2).

Our methodological approach did not allow for the quantification of glycan structures present on less than 1% of the identified peptides. Therefore, we can only conclude that this O-linked glycan is of low abundance in the E protein preparation. However, although this site is mainly non-glycosylated, our data imply that the glycan is present under certain conditions; the specific circumstances and significance of this glycan remain unknown.

**Figure 3:**
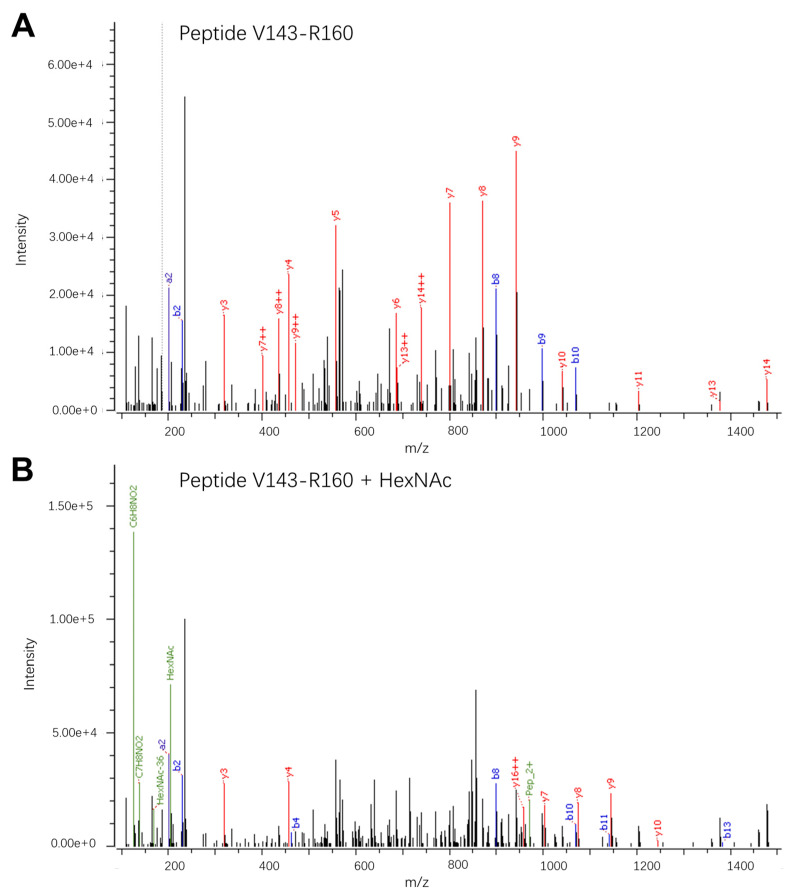
LC-MS/MS spectra showing the fragmentation patterns of the peptide V143-R160: VEPHTGDYVAANETHSGR, MW = 1938.8820. (**A**) Fragment ion spectrum of the non-glycosylated peptide; *m*/*z* = 647.3015; z = 3. (**B**) Fragment spectrum of the corresponding peptide carrying a single HexNAc modification; *m*/*z* = 714.9937; z = 3. The observed peptide fragments (b and y) are marked as well as the glycan oxonium ions observed in the low-*m*/*z* region of the fragment spectrum of the HexNAc-modified peptide.

Secondly, a confirmed O-linked glycan was identified on peptides covering amino acids V143-R160, which contain two potential sites for O-linked glycosylation (T147 and S158). Multiple glycoforms were observed for the V143-R156 peptide due to the presence of both N- and O-linked glycans. All sixteen glycoforms observed at relative abundances above 1% are listed in Table S1. Of these glycoforms, the peptide carrying a single N-acetylgalactosamine (GalNAc) was observed at 1.1% relative abundance. The presence of an O-linked GalNAc was confirmed by a high-intensity oxonium ion at *m*/*z* 126 during manual inspection of the ion spectra (Figure 3).

Since the site for O-linked glycosylation on peptides covering amino acids V143-R160 is close to the N-linked site N154, this glycan may impact viral entry, infectivity, or pathogenesis either independently or through interaction with the N-linked glycan. Additionally, this O-linked glycan is near one of the three identified B-cell epitopes (covering amino acids 163–180) [11]. Our group previously demonstrated that antibody reactivity to a peptide decorated with a single GalNAc varies depending on the site of the modification and that specific glycans can both enhance and reduce antibody reactivity [39,40].

**Figure 4:**
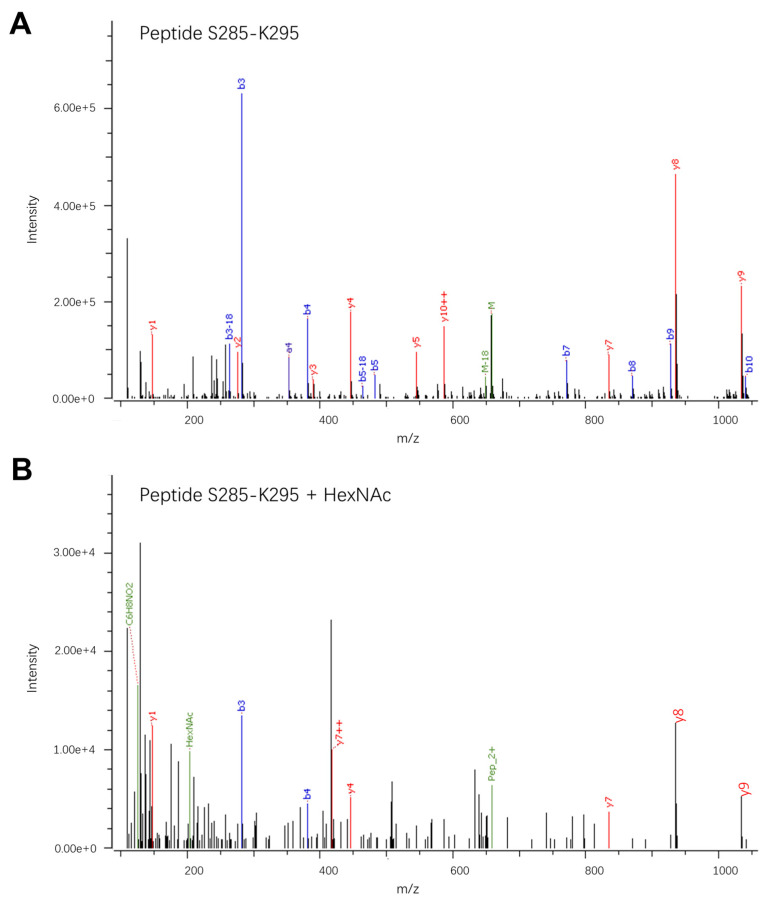
LC-MS/MS spectra showing the fragmentation patterns of the peptide S285-K295: SGHVTCEVGLEK, MW = 1314.6238 Da. (**A**) Fragment ion spectrum of the non-glycosylated peptide; *m*/*z* = 648.3; z = 2. (**B**) Fragment spectrum of the corresponding peptide carrying a single HexNAc modification; *m*/*z* = 506.9083; z = 3. The observed peptide fragments (b and y) are marked as well as the glycan oxonium ions observed in the low-*m*/*z* region of the fragment spectrum of the HexNAc-modified peptide.

The third identified O-linked glycan has two possible sites: S285 or T289. Manual inspection of the ion spectra revealed a single GalNAc on peptides covering amino acids S285-K296 (Figure 4). Similar to the O-linked glycan on sites T76, T81, or T90, this glycan is of low abundance, present on less than 1% of the analyzed peptides.

Next, we compared the amino acid sequences of 762 samples (European, Siberian, and Far-Eastern TBEV subtypes) deposited in GenBank, including 396 full-length protein E sequences classified as the European strain (Tables S2 and S3). As expected, the N-linked consensus site N154 was conserved in all samples, showing 100% identity across all 762 analyzed sequences. There was also a high degree of conservation among the amino acids identified as potential carriers of O-linked glycan structures. Specifically, T76, T90, and T289 showed 100% identity in all sequences, and T147 was in four cases replaced with S147, thereby still permitting O-linked glycosylation. T81, S158, and S285 showed 94.5%, 98.8%, and 99.5% identity, respectively, across all strains. Among European strains, all potential glycosites presented 100% identity, except for T81 (96.0%) and S158 (98.7%). In summary, it appears that most of the potential O-linked glycan sites are conserved among all analyzed TBEV protein E sequences. For each O-linked glycan identified, there is at least one potential site with 100% identity.

When comparing TBEV with 17 other tick-borne flaviviruses [41], the O-glycan site T76/T81/T90 showed 100% identity in at least one of these positions. The T147/S158 site showed 89% identity, with two tick-borne flaviviruses exhibiting amino acid substitutions at both the 147 and the 158 site, which would prevent O-linked glycosylation. The S285/T289 showed 100% identity, as all viruses had a serine or threonine at position 289. Altogether, these findings suggest a strong conservation of putative O-linked glycosylation in a wide range of tick-borne flaviviruses, including species that do not infect humans. To our knowledge, there are no reports of O-linked glycosylation on the E protein isolated from tick cells. Thus, we cannot rule out that the conservation of these glycan sites may be driven by O-linked glycosylation processes within ticks.

**Figure 5:**
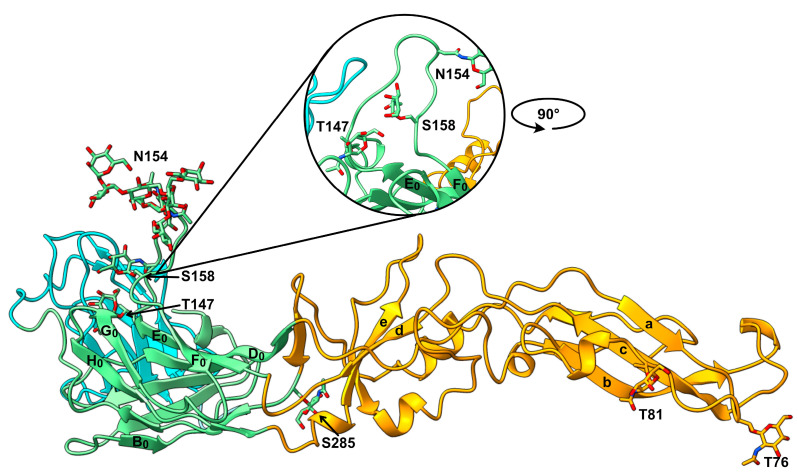
Structural overview of the N- and O-linked glycosylated E protein (PDB 7QRE), with the E 150-loop in open configuration. A representation of the most abundant oligomannose (Man6GlcNac2) is attached to N154. Single GalNAc monosaccharides are attached to T76, T81, T147, S158, and S285. The round inset shows the E 150-loop after rotating the model 90° counterclockwise. Selected β-strands are labeled in sequence order. Domain I: green; Domain II: orange; Domain III: blue.

To further investigate the potential impact of the O-linked glycans, we performed structural analysis of the E protein (PDB ID: 7QRE [9]) using the Glycoprotein Builder from GLYCAM Web [24]. Prior to analysis, the precursor membrane protein fragment and non-standard residues were removed in ChimeraX [23]. All potential sites for O-linked glycosylation, along with the single site for N-linked glycosylation, were tested by adding single GalNAc residues or the most frequently observed N-linked oligomannose structure (Man_6_GlcNac_2_) to N154, with the E 150-loop in its open configuration (Figure 5). In the model, sites T90 and T289 could not accommodate GalNAc residues due to a low calculated solvent-accessible surface area. Notably, T147 and S158 are part of the E 150-loop [9], and both sites can accommodate a GalNAc in this model.

**Figure 6:**
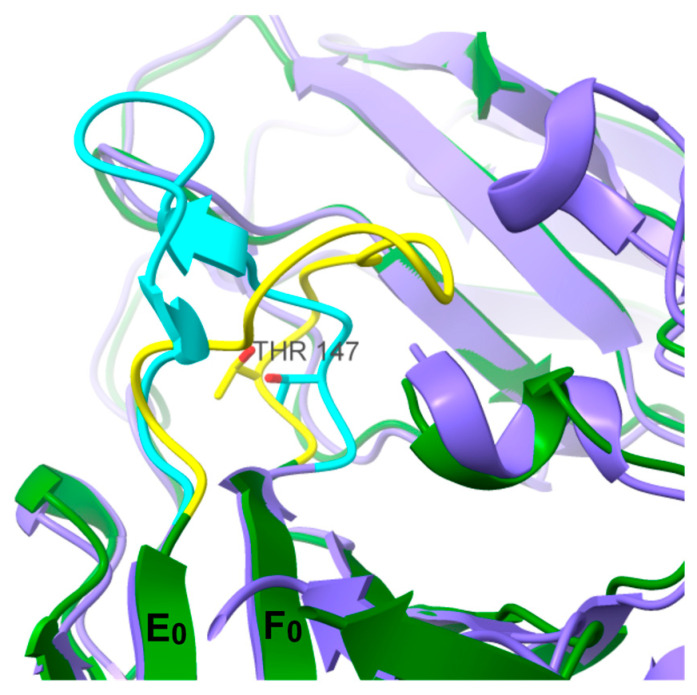
Superimposed non-glycosylated protein structures of PDB ID 1SVB and 7QRE; the proteins are colored green and purple, respectively, and residues 146–160, constituting the E 150-loop, are colored yellow and blue. The E 150-loop of 7QRE is kept open at acidic pH by interactions with the precursor to M protein (prM) while the yellow loop of 1SVB is in a closed conformation. The prM is not displayed in the figure. The location of T147 varies between the two models and could not be glycosylated in silico for 1SVB.

When analyzing the site occupancy of O-linked glycans within the E protein of PDB ID 1SVB [14,28], where the E 150-loop is in its closed conformation, the T147 site was unable to accommodate a GalNAc residue. Closer inspection of the E 150-loop suggests that its closed conformation would sterically hinder O-linked glycosylation at site T147 (Figure 6 *and* Figure S1).

Although only 1.1% of the peptides carried an O-linked glycan near N154 (at T147 and S158), and n even fewer carried O-linked glycans at the other positions within the E protein, this post-translational modification could still impact viral pathogenesis. Given the high number of viral particles present in the blood during TBEV viremia, a significant portion may harbor O-linked glycans [42]. Interestingly, sequence comparison revealed that T147 is highly conserved, with the threonine replaced by serine in only four cases, possibly indicating selective pressure to preserve O-linked glycosylation potential at this site. Additionally, predictions suggest that T147 is accessible for GalNAc addition only when the E 150-loop is in the open conformation. We propose three possible scenarios compatible with O-linked glycosylation at T147 or S158. (i) Early TGN glycosylation: Addition of a GalNAc residue at T147 occurs early in the TGN, potentially stabilizing the E 150-loop in an open configuration. In this scenario, the GalNAc would need to be cleaved before the E 150-loop closes in a pH-dependent manner [9,43]. (ii) Alternative site: Addition of a GalNAc residue occurs at site S158 which may or may not impact the E 150-loop function. (iii) We harvested the E protein from infected cells, and it cannot be ruled out that a proportion of the peptides come from misfolded proteins destined for degradation. Thus, the glycosylation that we observed at site T147 or S158 may be an artefact. At present, we cannot determine which scenario is most likely. However, the N-linked glycan at N154, located at the distal tip of the E 150-loop, plays an important role in viral infectivity and may affect cell tropism, including blood–brain barrier crossing [15,19]. The underlying mechanisms remain unclear, and further investigation into how the composition of the N-linked glycan and the presence of an adjacent O-linked glycan influence viral–host interaction is warranted.

The identified low-abundance O-linked glycans at sites T76, T81, or T90 and at sites S285 or T289 are within or close to ED II. This domain contains the highly conserved fusion loop (amino acids 98–110) [44], which mediates membrane fusion and dimer formation through interactions with the precursor to M protein (prM). It is also the primary target for cross-reacting antibodies [45,46,47]. Post-translational modifications, such as glycosylation of B-cell epitopes, can potentially alter antibody recognition. Since the glycans on viral proteins are synthesized by the host cell glycosylation machinery, they may be perceived as “self” by the immune system and therefore do not induce an immune response. Instead, these large, self-like glycans might physically hinder the binding of neutralizing antibodies due to physical hindrance, limiting the protective effect of the antibody epitopes [48]. In contrast, smaller glycans, such as a single GalNAc, can enhance antibody recognition. For instance, 70% of sera from patients infected with herpes simplex virus type 2 (HSV-2) contain IgG directed toward a heptamer within glycoprotein G, which carries a single GalNAc modification at T504. This serum reactivity is lost when the glycan moiety is absent [49].

Additionally, depending on the glycan modification site, individual sera can elicit diverse responses toward the same glycosylated epitope [39]. This suggests that glycosylation can influence antigen processing and presentation by antigen-presenting cells (APCs), resulting in a shifted immunodominance where the antibody repertoire is directed more strongly towards certain epitopes [50]. Moreover, many vaccines against TBEV are based on an inactivated virus produced in chick embryo fibroblasts (CEF), likely resulting in viral particles containing CEF glycan signatures. A recombinant vaccine, with a glycosylation profile significantly different from the actual glycosylation present on the viral particle, might confer lower protection than a vaccine with a matching glycosylation profile [51]. Therefore, the glycosylation profile should be carefully in the development of new vaccine candidates.

## 4. Conclusions

We conducted a detailed characterization of the glycan profile of the E protein and identified three novel O-linked glycans in addition to the previously described N-linked glycan. A comparison of E protein sequences from the European, Siberian, and Far-Eastern TBEV subtypes revealed a high degree of conservation at most potential sites for O-linked glycosylation as well as at the N-linked glycan site. Although the number of peptides carrying O-linked glycans is low, it is intriguing that two of these positions (T147 and S158) are located close to the E 150-loop and that putative addition of an O-linked glycan to T147 may regulate E 150-loop function.

## Data Availability

The original contributions presented in the study are included in the article/Supplementary material; further inquiries can be directed to the corresponding author.

## Supplementary Materials

The following supporting information can be downloaded at https://www.mdpi.com/article/10.3390/v16121891/s1: Figure S1: Location of the red oxygen atom on the side chain of T154 in the E-protein, with the protein shown in sphere presentation; Table S1: Observed glycopeptides of site N154 of TBEV strain F7203 grown in the human adenocarcinomic cell line A549; Table S2: Amino acid similarity at potential glycosylation sites within 762 protein E sequences of European, Siberian and Far-Eastern TBEV subtypes; Table S3: Amino acid similarity at potential glycosylation sites within 396 protein E sequences of European TBEV strains.
