# Supplementary material for: Identification of Three Novel O-Linked Glycans in the Envelope Protein of Tick-Borne Encephalitis Virus
Source: Viruses. 2024 Dec 8;16(12):1891. doi: 10.3390/v16121891 (PMC11680210; doi:10.3390/v16121891)
Supplement: Supplementary file 1 [file viruses-16-01891-s001.zip › viruses-3279365-supplementary.pdf]

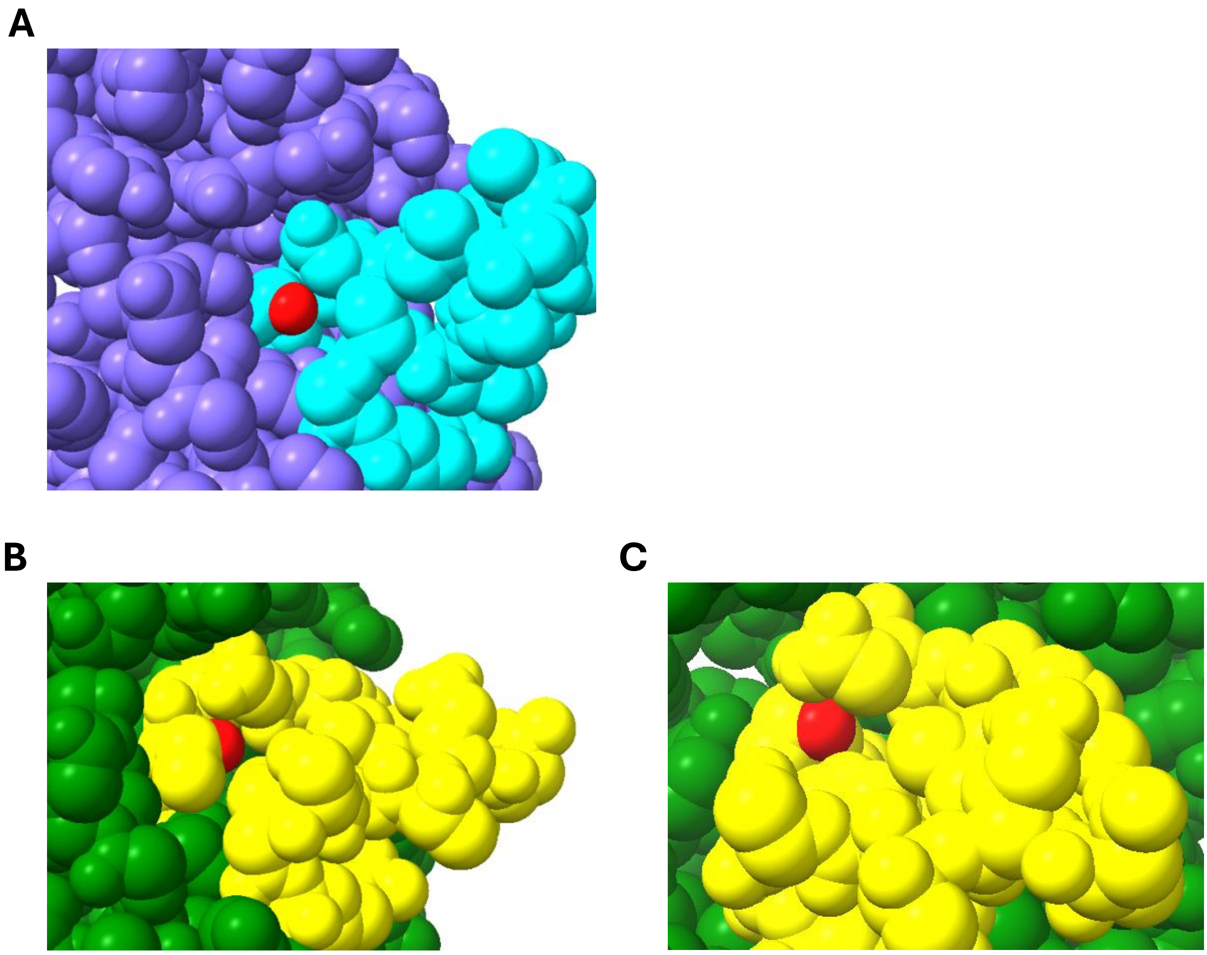

Figure S1. Location of the red oxygen atom on the side chain of T147 in the E-protein, with the protein shown in sphere presentation. **(A)** Image of PDB 7QRE with E-150 loop in open configuration, colored cyan. The rest of the protein is colored purple. **(B)** Image of PDB 1SVB from the same angle as panel A, with the E-150 loop in closed configuration, colored in yellow. The rest of the protein is colored green. **(C)** The same model as panel B, but rotated 60 degrees counterclockwise around the vertical axis to show the most favorable visual accessibility.

Supplementary Table S1. Observed glycopeptides of site N154 of TBEV strain F7203 grown in the human adenocarcinomic cell line A549. Percentage distribution is based on the precursor ion abundances of a singel injection. Peptides with a relative abundance of ≤ 1 % are excluded.

**Peptide sequence**	**Glycan Composition**	**Average of Theo. MH+ [Da]**	**Abundance (% of total observed)**	**Assigned glycan type**
[K].VEPHTGDYVAANETHSGR.[K]		1939.889	2.2	NG
[K].VEPHTGDYVAANETHSGR.[K]	HexNAc(1)	2244.508	1.1	O-linked
[K].VEPHTGDYVAANETHSGR.[K]	HexNAc(2)Hex(3)	2832.207	3.6	paucimannose
[K].VEPHTGDYVAANETHSGR.[K]	HexNAc(2)Hex(4)	2994.259	9.6	paucimannose
[K].VEPHTGDYVAANETHSGR.[K]	HexNAc(2)Hex(5)	3156.312	5.6	oligomannose
[K].VEPHTGDYVAANETHSGR.[K]	HexNAc(2)Hex(6)	3318.365	12.1	oligomannose
[K].VEPHTGDYVAANETHSGR.[K]	HexNAc(2)Hex(7)	3480.418	6.3	oligomannose
[K].VEPHTGDYVAANETHSGR.[K]	HexNAc(2)Hex(8)	3642.471	9.0	oligomannose
[K].VEPHTGDYVAANETHSGR.[K]	HexNAc(2)Hex(9)	3804.523	8.8	oligomannose
[K].VEPHTGDYVAANETHSGR.[K]	HexNAc(3)Hex(5)	3359.392	1.1	other*
[K].VEPHTGDYVAANETHSGR.[K]	HexNAc(3)Hex(5)Fuc(1)	3505.449	1.0	other*
[K].VEPHTGDYVAANETHSGR.[K]	HexNAc(3)Hex(6)	3521.444	2.9	other*
[K].VEPHTGDYVAANETHSGR.[K]	HexNAc(4)Hex(5)	3562.471	2.6	complex
[K].VEPHTGDYVAANETHSGR.[K]	HexNAc(4)Hex(5)Fuc(1)	3708.529	21.3	complex
[K].VEPHTGDYVAANETHSGR.[K]	HexNAc(4)Hex(5)Fuc(1)NeuAc(1)	3999.624	4.6	complex
[K].VEPHTGDYVAANETHSGR.[K]	HexNAc(5)Hex(6)Fuc(1)	4073.661	1.7	complex

NG = non glycosylated, * the category "other" could include both N-linked glycans of hybrid type, and a combination of N-linked and O-linked glycoforms present on the same peptide.

Supplementary Table S2. Amino acid similarity at potential glycosylation sites within 762 protein E sequences of European, Siberian and far eastern TBEV subtypes.

Amino acid position	Similarity	Substitutions
N154	100%	
T76	100%	
T81	94.5%	17 I81 (n=17), V81 (n=7), A81 (n=18)
T90	100%	
T147	99.5%	S147 (n=4)
S158	98.8%	N158 (n=6), K158 (n=3)
S285	99.5%	K285 (n=1), G285 (n=3)
T289	100%	

Supplementary Table S3. Amino acid similarity at potential glycosylation sites within 396 protein E sequences of European TBEV strains.

**Amino acid position**	**Similarity**	**Substitutions**
N154	100%	
T76	100%	
T81	96.0%	I81 (n=16)
T90	100%	
T147	100%	
S158	98.7%	N158 (n=5)
S285	100%	
T289	100%	
